# Expected reward modulates encoding-related theta activity before an event

**DOI:** 10.1016/j.neuroimage.2012.07.064

**Published:** 2013-01-01

**Authors:** Matthias J. Gruber, Andrew J. Watrous, Arne D. Ekstrom, Charan Ranganath, Leun J. Otten

**Affiliations:** a Institute of Cognitive Neuroscience, University College London, 17 Queen Square, London WC1N 3AR, UK; b Center for Neuroscience, University of California at Davis, 1544 Newton Court, Davis, CA 95618, USA

**Keywords:** Prestimulus activity, Memory encoding, Reward, Theta, Anticipation, Long-term memory

## Abstract

Oscillatory brain activity in the theta frequency range (4–8 Hz) before the onset of an event has been shown to affect the likelihood of successfully encoding the event into memory. Recent work has also indicated that frontal theta activity might be modulated by reward, but it is not clear how reward expectancy, anticipatory theta activity, and memory formation might be related. Here, we used scalp electroencephalography (EEG) to assess the relationship between these factors. EEG was recorded from healthy adults while they memorized a series of words. Each word was preceded by a cue that indicated whether a high or low monetary reward would be earned if the word was successfully remembered in a later recognition test. Frontal theta power between the presentation of the reward cue and the onset of a word was predictive of later memory for the word, but only in the high reward condition. No theta differences were observed before word onset following low reward cues. The magnitude of prestimulus encoding-related theta activity in the high reward condition was correlated with the number of high reward words that were later confidently recognized. These findings provide strong evidence for a link between reward expectancy, theta activity, and memory encoding. Theta activity before event onset seems to be especially important for the encoding of motivationally significant stimuli. One possibility is that dopaminergic activity during reward anticipation mediates frontal theta activity related to memory.

## Highlights

► Prestimulus theta activity predicts subsequent memory formation. ► Theta activity is also sensitive to reward expectancy. ► Is there a relationship between theta, expected reward, and memory formation? ► Prestimulus theta only predicted later memory performance for high reward trials. ► Prestimulus theta seems crucial for encoding of motivationally significant stimuli.

## Introduction

There is growing evidence that, in addition to brain activity that is evoked by an event, brain activity patterns that precede the onset of an event can contribute to memory performance. Several functional magnetic resonance imaging (fMRI) and magneto-/electroencephalography (M/EEG) studies have shown that activity just before initially encountering a new stimulus can predict whether that stimulus will be remembered during a later memory test (Adcock et al., 2006; Bollinger et al., 2010; Fell et al., 2011; Galli et al., 2011; Gruber and Otten, 2010; Guderian et al., 2009; Mackiewicz et al., 2006; Otten et al., 2006, 2010; Padovani et al., 2011; Park and Rugg, 2010; Uncapher et al., 2011). In particular, M/EEG studies have shown that oscillatory activity in the theta (4–8 Hz) frequency band prior to the onset of a study or test item is enhanced on trials for which the item is subsequently remembered (Addante et al., 2011; Fell et al., 2011; Guderian et al., 2009). These findings suggest that theta activity can set the stage for effective encoding and retrieval. However, it is largely unknown what factors influence such prestimulus theta activity.

Studies in rodents suggest that reward may be one factor that contributes to theta activity (Benchenane et al., 2010; van Wingerden et al., 2010). For instance, van Wingerden et al. (2010) found that firing of neurons in the orbitofrontal cortex of rats showed increased theta phase locking during anticipation of a reward. In humans, reward motivation has been linked to successful memory encoding (Adcock et al., 2006; Wittmann et al., 2005), suggesting that there might be a relationship between reward expectancy, anticipatory theta activity, and memory encoding in humans.

To test this possibility, we took advantage of a data set previously only considered in terms of event-related potentials (Gruber and Otten, 2010). Oscillatory theta power was extracted from scalp-recorded electrical brain activity obtained during intentional memorization of words (cf. Adcock et al., 2006). Each word was preceded by a low or high reward cue, which indicated the amount of money that would be earned if the word was remembered in a later recognition test. Rewards were delivered at the end of the experiment. The analyses focused on theta power in the interval between the reward cue and word onset. The question of interest was whether theta activity before word onset (hereafter referred to as ‘prestimulus’ activity) would predict subsequent memory of the word and, if so, whether this activity would differ between low and high reward cues.

## Methods

### Participants

The experimental procedures were approved by the University College London Research Ethics Committee. The analyses reported here are based on a subset of twenty participants in Gruber and Otten (2010) who had at least twelve artifact-free remembered and forgotten trials in either the high or low reward condition (for details, see the Statistical analyses section). Participants (mean of age 24 years; range of 19–33 years; 7 men) were right-handed, had normal or corrected-to-normal vision, and reported not to have had any neurological or psychiatric illnesses. All participants provided informed written consent before participating.

### Procedure

**Fig. 1:**
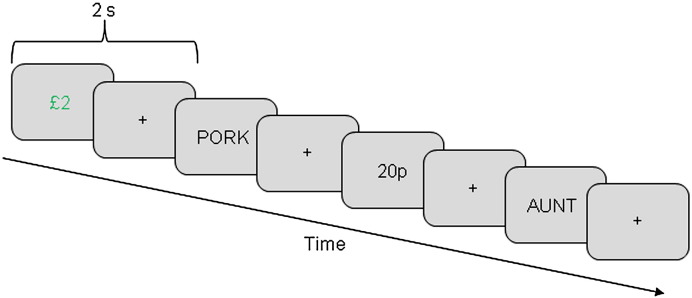
Experimental procedure of the encoding phase. Each word (e.g. ‘pork’ or ‘aunt’) was preceded by either a low or a high reward cue (‘20p’ written in black or ‘£2’ written in green, respectively). The reward cue indicated the amount of money that would be received if the upcoming item was remembered in a later recognition test. The interval between cue and word was constant (i.e. 2 s) and was used for the analyses of encoding-related theta activity.

Participants completed an intentional memorization task (Fig. 1), followed by a recognition memory test after a delay of about 15 min. Stimuli consisted of a series of 4–8 letter visually-presented words with a written frequency of 1–30 per million (Kučera and Francis, 1967). Each study trial commenced with the presentation of a low (‘20p’ written in black) or high (‘£2’ written in green) monetary reward cue 2 s before word onset. The cue was presented for a duration of 1 s and was replaced by a fixation cross for the remaining 1 s of the cue-word interval. The study sequence consisted of 120 low and 120 high randomly intermixed reward cues. Words were shown for 0.5 s followed by a fixation cross until the next cue. The time between word onset until the next cue varied randomly between 4 and 5.5 s. Participants were told that the cue represented the amount of additional money they would receive at the end of the experiment if they successfully recognized the upcoming word in the later memory test. Participants received 5% of their total earnings on top of the hourly reimbursement rate. In addition to memorizing the words, participants also had to indicate whether the first and last letters of a word were in alphabetical order to ensure attention was paid to all words.

All 240 studied words were shown again in the recognition memory test, intermixed with 120 new words. Each test trial commenced with the presentation of a neutral warning stimulus (‘!’) for 1 s, followed by the presentation of the test word for 0.5 s. The interval between successive warning stimuli varied randomly between 4 and 5.5 s. The memory test incorporated a five-way recognition confidence judgment (Yonelinas et al., 2005). Participants pressed one of five buttons to indicate that they (i) recollected specific details about the word's initial occurrence (a ‘remember’ response), (ii) were confident about the word's earlier occurrence without further details (a ‘confident old’ response), (iii) thought that they recognized the word (an ‘unconfident old’ response), (iv) thought the word was new to the experiment (‘unconfident new’), or (v) were confident that the word had not been presented earlier (a ‘confident new’ response). The time–frequency analyses focused on the comparison between studied items later confidently judged as old (i.e. given a ‘remember’ or ‘confident old’ response) and items later incorrectly judged as new (i.e. given an ‘unconfident new’ or ‘confident new’ response). Insufficient trials were available to split these categories further. Rewards were provided for any correctly recognized old word, regardless of the type of judgment associated with the decision. False alarms attracted a £2.50 penalty to discourage participants from saying ‘old’ all the time.

### EEG acquisition and time–frequency analyses

Electrical brain activity was recorded during the memorization task with sintered silver/silver-chloride electrodes attached to 34 scalp sites (montage 10 at www.easycap.de/easycap/e/electrodes/13_M10.htm) referenced to the midfrontal site Fz. Vertical and horizontal eye movements were recorded from electrodes above and below the right eye and at the outer canthi, respectively. Signals were digitized at 500 Hz and filtered with a bandpass between 0.01 and 35 Hz.

The EEGLAB toolbox (Delorme and Makeig, 2004) was used to pre-process the EEG data and conduct time–frequency analyses. The continuous EEG was high-pass filtered with a cut-off at 0.5 Hz and divided into epochs ranging from 600 ms before cue onset until 4.2 s thereafter. This epoch length was chosen to derive time–frequency information for the entire 2 s cue-word interval and 1.6 s after word onset. Inclusion of the 600 ms periods at the beginning and end of the epochs was necessary to avoid edge effects in the time periods of interest. Epochs were baseline-corrected in the time domain using the mean signal in the 600 ms period before cue onset. Trials in which EEG activity exceeded more than three standard deviations from the mean on that electrode or five standard deviations across all electrodes were excluded from the analyses. Blinks and eye movements were removed via independent component analysis (Bell and Sejnowski, 1995; Delorme and Makeig, 2004), which has been shown to be an effective way to eliminate such artifacts from EEG (e.g. Hoffmann and Falkenstein, 2008). A final check was performed manually and any trials that contained further muscle artifacts, amplifier saturations or incorrect alphabetic responses were excluded from the analyses. The data were then re-referenced to the average of the left and right mastoids. The online reference at the midline frontal site Fz was re-instated and used as a scalp site of interest.

Time–frequency analyses were conducted using Morlet wavelets (Percival and Walden, 1993) with 4 cycles and a sliding time window which was moved in 10 ms increments in the 0–3.6 s interval. This computation was done in steps of 1 Hz from 4 to 12 Hz. The analyses reported here did not employ a baseline in the frequency domain. Because the focus here was on differences in oscillatory power between trial types, this step was not necessary (cf. Addante et al., 2011; Fell et al., 2011; Guderian, et al., 2009; Mazaheri et al., 2009). An additional analysis using mean frequency power across the epoch as a baseline led to the same conclusions as those reported here. For each participant, EEG data during study trials were binned according to the type of reward cue (low vs. high) and subsequent memory performance (remembered vs. forgotten). Words were classified as remembered when they were given a ‘remember’ or ‘confident old’ judgment and as forgotten when they received an ‘unconfident new’ or ‘confident new’ judgment. The mean numbers of remembered and forgotten trials were, respectively, 33 and 42 in the low reward condition and 56 and 24 in the high reward condition. Activity that differed between later remembered and later forgotten items was taken to be important for successful encoding (Paller and Wagner, 2002). The analyses focused on theta power. Possible encoding-related differences in inter-trial theta phase coherence were considered in a separate analysis, but no effects were observed.

### Statistical analyses

In total, twenty participants entered the analyses who had at least twelve artifact-free remembered and forgotten trials in either reward condition. To statistically test the difference in encoding-related activity between high and low reward conditions, the analyses included twelve participants with a sufficient number of trials in both reward conditions. For consistency, all key analyses are reported for these twelve participants. However, we also repeated the analyses on encoding-related activity in each reward condition using the maximum number of participants who had sufficient remembered and forgotten trials in either reward condition (18 and 14 participants in the high and low reward conditions, respectively). The effects found in the analyses that used the maximum number of participants did not differ from those reported for the 12 participants with sufficient trials in both reward conditions.

Across-trial permutation tests were used to test for the statistical significance of encoding-related theta power in the cue-word interval. To that end, the 2 s interval was collapsed into ten time windows of 200 ms each. For each window, mean power between 4 and 6 Hz (where encoding-related activity was most evident) was calculated for each electrode, participant, and trial. The permutation tests were performed in each time window to reveal electrode clusters with significant encoding-related theta activity during that period of time. The initial analyses focused on the interaction between encoding-related activity across low and high reward conditions. Significant effects were followed with permutation tests in each reward condition to assess significant encoding-related activity in each. For completeness, we also computed encoding-related activity collapsed across reward conditions (cf. Guderian et al., 2009).

The permutation tests were adopted from Blair and Karniski (1993; for similar approaches see Addante et al., 2011; Hanslmayr et al., 2009; Maris and Oostenveld, 2007; Staudigl et al., 2010). First, two-tailed *t* tests were run on the data from each electrode site on the relevant two conditions. Second, the trials were randomized and split in half into two pseudo conditions. The *t* tests were then rerun on the pseudo conditions. The second step was repeated 1000 times and the obtained pseudo *t* values were sorted in ascending order, yielding a distribution of 1000 pseudo *t* values. In the final step, the two tails from the pseudo *t* value distribution were used as the critical *t* values to reject the null hypothesis that the actual difference between the two experimental conditions was significant. Using an alpha level of 0.05 and 1000 permutations, the 25th and 975th values represent the critical *t* values. This procedure was conducted for all 32 scalp electrodes and would therefore be expected to lead to a Type 1 error on 1.6 electrodes (32 ∗ 0.05) per time window assuming independence across sites. We therefore only considered effects that spanned across two electrodes, restricting ourselves to neighboring electrodes because volume conduction would be expected to lead to effects that are spatially close. To further reduce the chance of noise-related effects, we only interpreted significant clusters that spanned across two consecutive time bins. This allowed the measurement of two rather than one theta cycle, increasing robustness of the estimation. The data were collapsed across significant time windows and another permutation test was run on this extended window (for a similar approach, see Pastötter et al., 2011). For simplicity, we only report the effects in the extended time windows.

## Results

### Task performance

**Fig. 2:**
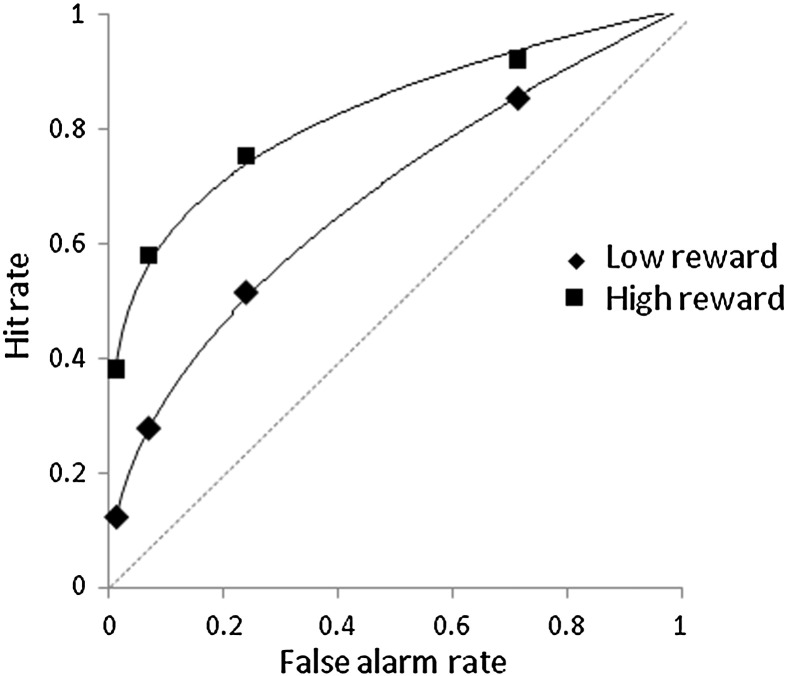
Recognition memory performance. Receiver operating characteristic (ROC) curves for words in the low and high reward conditions.

For consistency with the EEG analyses, behavioral results are reported for the subset of 12 participants who had sufficient numbers of trials in each of the critical bins for both the high and low reward conditions (see the Statistical analyses section for details). The results indicated that reward cues influenced performance on the recognition memory test but not the encoding task. The accuracy with which alphabetic judgments were made during encoding did not differ across reward conditions (0.92 and 0.90 in the high and low reward conditions, respectively, *t*_11_ = 1.64, *p* = 0.129). Similarly, the speed of alphabetic judgments did not differ (1590 and 1565 ms in the high and low reward conditions, respectively, *t*_11_ = 0.53, *p* = 0.609). However, discrimination accuracy during the recognition memory test, computed as the difference between the confident hit rate (proportion of old words correctly given a ‘remember’ or ‘confident old’ judgment) and false alarm rate (proportion of new words incorrectly judged as old; Snodgrass and Corwin, 1988), was better for words on high reward trials (0.48 and 0.24 in the high and low reward conditions, respectively, paired-sample *t*_11_ = 7.00, *p* < 0.001). Across participants, memory performance in the high reward condition correlated positively with performance in the low reward condition (*r* = 0.734, *p* = 0.007). This suggests that participants who performed better in the high reward condition did so because their memory was on the whole better, and not because they were more successful at ignoring low reward words. For completeness, we also computed receiver operating characteristic (ROC) curves and these are presented in Fig. 2.

### Encoding-related theta activity

**Fig. 3:**
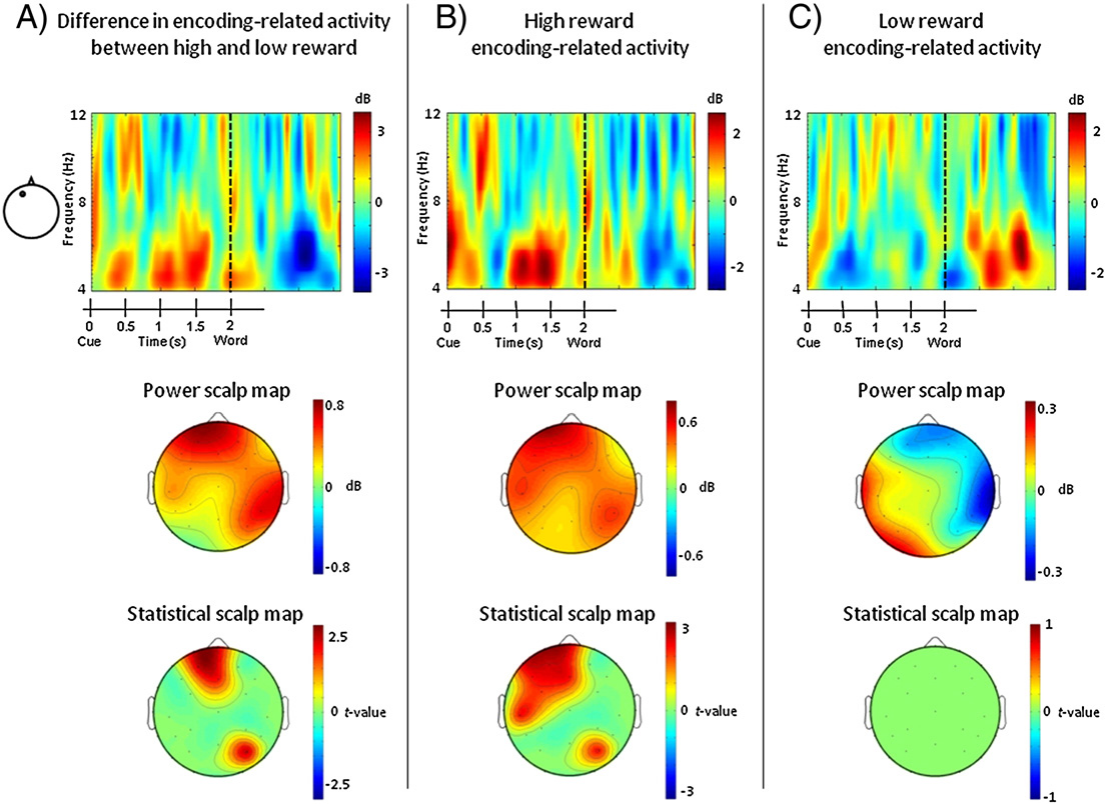
Encoding-related theta activity before word onset. Top row: Time–frequency representations of the difference in oscillatory power at a frontal scalp site between words that were later confidently recognized or forgotten (i.e. encoding-related activity). The frontal electrode represents site 50 from montage 10, www.easycap.de/easycap/e/electrodes/13_M10.htm, equivalent to Fp1 in the 10/10 system; the data were spatially smoothed for display purposes. The time–frequency representations display the difference in encoding-related activity between the low and high reward conditions (A), encoding-related activity in the high reward condition (B), and encoding-related activity in the low reward condition (C). A prestimulus subsequent memory effect in the theta frequency band can be seen in the middle of the cue-word interval in the high reward condition only. Middle row: Scalp maps depicting the location of encoding-related theta activity in the 1–0.4 s interval before word onset (i.e. 1–1.6 s interval after cue onset). The maps depict encoding-related theta power between the low and high reward conditions (A), the high reward condition (B), and the low reward condition (C). Bottom row: Statistical scalp maps corresponding to the power scalp maps displayed in the middle row. Instead of the data, the maps show the statistics of the permutation tests on encoding-related theta power in the 1–0.4 s interval before word onset. The color coding represents the value of the *t* statistics where significant differences were found (*p* < 0.05). A frontal prestimulus subsequent memory effect in the theta frequency band is only evident in the high reward condition.

**Fig. 4:**
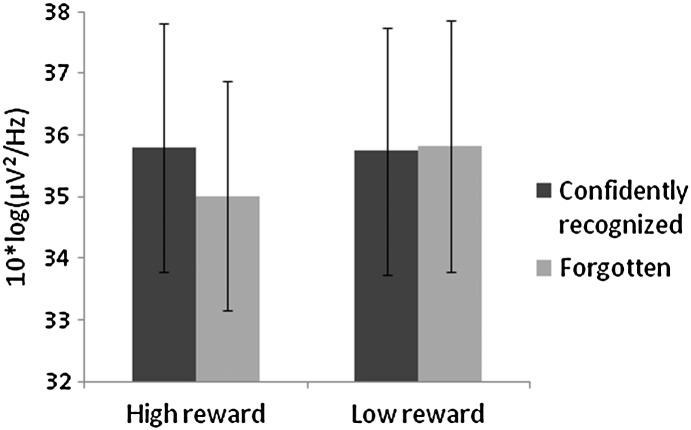
Absolute theta power preceding words that were later remembered versus forgotten in the low and high reward conditions. Values represent power averaged across the 1–0.4 s interval before word onset at site 50 from montage 10, www.easycap.de/easycap/e/electrodes/13_M10.htm, equivalent to Fp1 in the 10/10 system.

Our primary interest concerned the influence of reward cues on prestimulus oscillatory theta power and successful memory formation. The initial analyses therefore assessed differences in encoding-related theta activity (i.e. differences in theta power between later remembered and forgotten trials) between the low and high reward conditions in the cue-word interval. The analyses encompassed the entire 2 s in between the onset of the reward cue and the onset of the to-be-encoded word. Significant differences were found between 1 and 1.6 s after cue onset (i.e. between 1 and 0.4 s before word onset) on a cluster of frontal electrode sites (Fig. 3A). Follow-up tests in each reward condition indicated that the interaction arose because theta power differed between remembered and forgotten items in the high but not low reward condition. In the high reward condition, theta power differed significantly in the 1–0.4 s interval before word onset depending on whether the word was later remembered or forgotten (Fig. 3B). In the low reward condition, by contrast, no significant differences emerged before word onset as a function of subsequent memory performance (Fig. 3C). When the maximum numbers of participants were used for the follow-up analyses in the high and low reward conditions (18 and 14 participants, respectively), the same effects were found. Fig. 4 shows the pattern of absolute theta values for remembered and forgotten words in the two reward conditions. Permutation tests on the difference in theta power between low and high reward trials irrespective of later memory performance did not reveal reward-related effects in the cue-word interval.

**Fig. 5:**
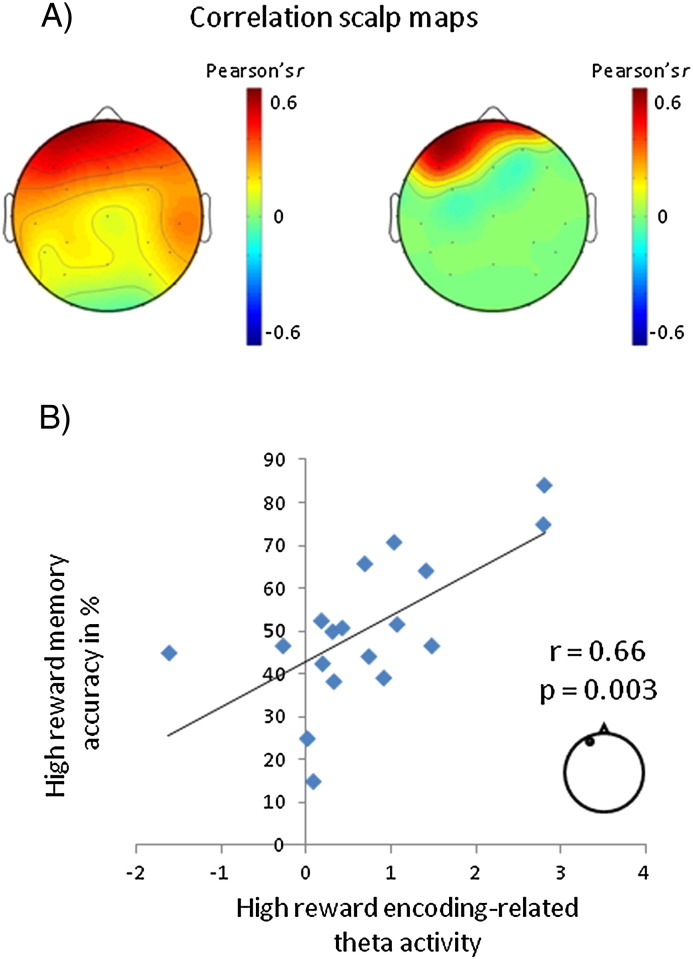
Relationship between prestimulus encoding-related theta activity and memory performance. (A) Representation of the correlation coefficients (Pearson's *r*) at each scalp site. The scalp maps show the correlation between participants' memory accuracy for confidently recognized high reward words and their prestimulus subsequent memory effects (theta power averaged over the 1–0.4 s interval before word onset). The scalp map on the left displays all *r* values; the map on the right only those that were significant (*p* < 0.05). For this analysis, all 18 participants were included that had at least 12 trials each for later remembered and forgotten words in the high reward condition. (B) Scatterplot of participants' memory accuracy for confidently recognized high reward words and prestimulus subsequent memory effects (averaged over the 1–0.4 s interval before word onset) at a frontal scalp location. Each dot represents the subsequent memory effect from one participant. As in the previous figures, the frontal scalp location represents site 50 from montage 10, www.easycap.de/easycap/e/electrodes/13_M10.htm, equivalent to Fp1 in the 10/10 system. As in (A), the plot includes all 18 participants with at least 12 remembered and forgotten trials in the high reward condition.

To further substantiate the role of prestimulus theta power in the high reward condition in effective encoding, we performed an across-subject correlation between a person's overall memory performance and the size of their prestimulus subsequent memory effect. The difference in theta power between later confidently recognized and later forgotten high reward words in the 1–0.4 s interval before word onset was correlated with participants' discrimination accuracy for confidently recognized high reward words (Fig. 5). We conducted this analysis for all 32 scalp electrodes separately and included all 18 participants who had at least 12 remembered and forgotten high reward words. The analyses showed that encoding-related theta power before word onset correlated positively with the ability to discriminate between old and new words. This correlation was specific to a cluster of frontal electrodes, which strongly resembled the left frontal locus of the encoding-related activity observed in the high reward condition (Fig. 5A). A scatterplot depicting the relationship between the prestimulus subsequent memory effect in theta power and recognition accuracy for high reward trials is shown for electrode Fp1 in Fig. 5B. None of the participants had theta subsequent memory effects or memory accuracies whose *z* score fell above or below 2.5 standard deviations of the mean. The correlations are thus not driven by outliers.

Subsequent memory effects irrespective of reward condition were assessed by collapsing encoding-related theta activity across the low and high reward conditions (cf. Guderian et al., 2009). This analysis revealed a significant increase in theta power over frontal scalp sites between 0 and 0.4 s after cue onset. However, permutation tests in each reward condition only showed this effect in the high reward condition whereas the effect did not emerge when the two conditions were directly contrasted. The effect was therefore not interpreted.

## Discussion

The aim of the study was to assess the effects of reward anticipation and EEG oscillations in the theta band on verbal memory encoding. We found that oscillatory theta power before word onset differed depending on whether the word was confidently recognized or forgotten in a later memory test. Crucially, theta activity only modulated memory performance when learning took place in the prospect of a high monetary reward. For low reward cues, theta activity before word onset did not predict later memory performance. Additionally, the larger the encoding-related theta power in anticipation of high reward words, the better was a participant's ability to discriminate between old and new information later on. These results suggest that reward anticipation facilitates memory encoding via oscillatory power in the theta frequency range before an event is even perceived. These findings provide a link between two disparate literatures — one linking theta oscillations to memory, and another linking theta to reward anticipation.

### Encoding-related prestimulus theta oscillations

The present results add to accumulating evidence linking fluctuations in theta activity prior to stimulus encoding and successful memory formation. For instance, Guderian et al. (2009) demonstrated that theta power increased around 200 ms before the presentation of words that were later freely recalled, relative to words that were not recalled. This theta enhancement was observed regardless of whether participants encoded an upcoming item in a semantic or non-semantic manner, indicating that the prestimulus effect was not related to preparation of the specific processes to be engaged following stimulus onset. Source estimation analyses indicated that the theta effect was consistent with generators in the medial temporal lobes. In line with the results of Guderian et al. (2009), Fell et al. (2011) reported a link between prestimulus activity in the human hippocampus and memory formation. Using intracranial recordings from the medial temporal lobe during performance of a continuous recognition test, Fell et al. (2011) found that low frequency power preceding words encountered for the first time in a continuous recognition test was predictive of whether the words would be accurately recognized upon re-occurrence. This was most evident for theta power in the hippocampus and for alpha power in the adjacent rhinal cortex. Finally, Rutishauser et al. (2010) showed that hippocampal spike-field coherence in the theta frequency range increased for words that were later highly confidently recognized. The relationship between spike-field coherence and subsequent memory performance was evident prior to word onset, suggesting that anticipatory theta activity may influence the activity of hippocampal neurons during stimulus encoding. This result is consistent with models which suggest that theta rhythms may play a critical role in influencing the timing of memory-related processes in the hippocampus and interactions between the hippocampus and other brain regions (Düzel et al., 2010; Hasselmo, 2005; Klimesch et al., 2008; Nyhus and Curran, 2010).

The prestimulus theta studies described above (Fell et al., 2011; Guderian et al., 2009; Rutishauser et al., 2010) suggest a link between anticipatory theta oscillations and memory encoding, but they do not provide much evidence regarding the factors that might drive changes in theta activity in anticipation of an upcoming item. The present results provide new evidence demonstrating that reward might be one such factor. That is, we found that prestimulus theta activity was related to successful encoding primarily during anticipation of a high reward. Interestingly, our finding converges, at least in part, with a recent study by Cohen et al. (2012) that also suggests a role of frontal scalp-recorded EEG in reward anticipation. Cohen et al. (2012) simultaneously recorded scalp and intracranial EEG from the nucleus accumbens when patients anticipated an upcoming reward. Spectral Granger causality analyses suggested that frontal scalp-recorded oscillatory power that was most prominent in the theta and delta frequency bands directly influenced activity in the nucleus accumbens. The authors found a stronger relationship between frontal scalp-related activity and nucleus accumbens when a high reward was anticipated. The results might be compatible with our finding suggesting that increased scalp-recorded frontal theta plays an important role in reward anticipation, thereby influencing memory formation.

### Possible mechanisms of theta oscillations in reward anticipation

Although we cannot make inferences about the intracerebral sources underlying the prestimulus frontal theta effect we observed, studies in animal models suggest some possibilities that can be investigated in future studies. Results from studies of rats and monkeys indicate that midbrain dopamine neurons show increased firing during anticipation of rewards (Fiorillo et al., 2003; Ljungberg et al., 1992; Schultz, 1998, 2002; Tabuchi et al., 2000) and, consistent with these data, human fMRI studies have shown that midbrain activation is increased during reward anticipation (Ballard et al., 2011; Carter et al., 2009; Knutson and Cooper, 2005; Knutson et al., 2001; O'Doherty, 2004; Schott et al., 2008). Furthermore, results from several fMRI studies suggest that activity in the dopaminergic midbrain might mediate the effect of rewards on memory encoding (Adcock et al., 2006; Bunzeck et al., 2012; Wittmann et al., 2005, 2008). For instance, an fMRI study by Adcock et al. (2006) showed that activity in the ventral tegmental area was larger following high reward cues, as compared to low reward cues. Furthermore, activity this region and in the hippocampus on high reward trials was enhanced prior to the onset of scenes that were successfully encoded. The findings by Adcock et al. (2006) indicate that the relationship between memory and reward already takes place before the onset of upcoming to-be-encoded information.

It is possible that dopaminergic activity during reward anticipation influences frontal theta activity. Consistent with this idea, Benchenane et al. (2010) showed that in rats dopamine modulates theta coherence between the medial prefrontal cortex and the hippocampus during the learning of new rules. When dopamine was administered to the medial prefrontal cortex of anesthetized rats, theta coherence between the prefrontal cortex and the hippocampus increased. A more direct link between reward and theta coherence was demonstrated by van Wingerden et al. (2010), who found that firing of neurons in the orbitofrontal cortex of rats showed increased theta phase locking during anticipation of a reward. Importantly, theta phase locking was only evident when specific reward associations were learned and not if established reward associations were changed. Finally, Fujisawa and Buzsáki (2011) recently showed coherent theta oscillations in the prefrontal cortex, hippocampus, and ventral tegmental area in rats during a memory task. The studies of Benchenane et al. (2010), van Wingerden et al. (2010), and Fujisawa and Buzsáki (2011) might provide some indirect hints about the potential mechanism underlying the observed relationship between reward expectancy, theta activity, and memory encoding. An important direction for future research will be to clarify whether dopaminergic activity, in particular, might mediate the relationship between frontal theta and memory.

If theta activity affects encoding via reward-related processes, it might be expected that theta should on the whole be larger during high than low reward trials. This is not what we observed. Even though theta before high reward words differed depending on later memory performance to the words, absolute power in the theta range was on average the same during high and low reward trials. It is worth noting in this respect that, in contrast to previous studies (Cohen et al., 2012; Fujisawa and Buzsáki, 2011; van Wingerden et al., 2010), rewards were not actually delivered during the encoding task in the present experiment. Participants received the money they earned at the end of the experimental session. It is thus possible that general theta increases only occur during the anticipation of an immediate reward. One reason for this might be that in the absence of immediate rewards, participants continue to rehearse high reward words during low reward trials. This would be expected to affect reward-related theta but not encoding efficacy. Low reward words could not have been ignored completely because of the requirement to make an alphabetic decision on all words. However, this leaves sufficient opportunity for the continuing rehearsal of high reward words to maximize later reward.

Alternatively, the proposed influence of reward on theta and encoding might be indirect. For example, rewards may encourage the engagement of a motivational context that aids later encoding, and prestimulus theta might reflect this context rather than reward-related processes per se. Such an explanation is in line with studies suggesting a link between encoding-related theta activity and item-context binding (Hanslmayr et al., 2009; Summerfield and Mangels, 2005; Tort et al., 2009).

A final issue to consider is the time course of the theta activity we observed before word onset. Encoding-related activity started in the middle of the cue-word interval, at around 1 s after cue onset, and finished shortly before word onset. This time course is consistent with several other studies that have used M/EEG to identify encoding-related activity before an event (Fell et al., 2011; Galli et al., 2011; Gruber and Otten, 2010; Otten et al., 2010; Padovani et al., 2011). The time at which prestimulus activity occurs varies somewhat across studies. While the above studies show activity in the middle of the cue-event interval similar to what we observed here, others show encoding-related differences immediately before event onset (Guderian et al., 2009; Otten et al., 2006). Prestimulus activity toward the middle and the end of a cue-event interval is perhaps better thought of as reflecting processes that lead up to the presentation of the to-be-encoded event rather than processes specifically related to the prestimulus cues. The exact functional role of encoding-related activity before word onset is not possible to discern from the present data alone, however. An important methodological point is that oscillatory effects in the middle of the cue-event interval, such as those observed here, cannot easily be explained by a smearing of activity that actually occurs after event onset. Future work employing cue-event intervals of varying durations may shed further light on the nature of prestimulus activity by assessing whether encoding-related activity retains its timing to the occurrence of the cue or event.

### Conclusion

In conclusion, our findings indicate a link between reward anticipation, theta activity, and memory formation. Because theta and dopamine are seen to play an important role in synaptic plasticity and learning (Düzel et al., 2010; Hasselmo, 2005; Lisman et al., 2011), further research on the relationship between reward and theta may lead to important insights into successful long-term memory functioning.
